# Risk factors and new inflammatory indicators of deep vein thrombosis after adult patella fractures

**DOI:** 10.3389/fsurg.2022.1028542

**Published:** 2022-11-02

**Authors:** Shuo Diao, Jingqiao Li, Jianyong Zhao, Dong Wang, Hanzhou Wang, Xiaopei Xu, Junlin Zhou

**Affiliations:** ^1^ Department of Orthopedic Surgery, Beijing Chaoyang Hospital, Capital Medical University, Beijing, China; ^2^ Department of Orthopedic Surgery, Hebei Jing-Xing Xian Hospital, Shijiazhuang, China; ^3^ Department of Hand Surgery, Cangzhou Hospital of Integrated Traditional and Western Medicine of Hebei Province, Cangzhou, China

**Keywords:** risk factor, inflammatory indicators, deep vein thrombosis, patella fracture, platelet-to-lymphocyte ratio (PLR)

## Abstract

**Objective:**

This study aimed to investigate the association between new inflammatory indicators at admission and the occurrence of preoperative deep vein thrombosis (DVT) in patients with patella fractures.

**Methods:**

A retrospective analysis of the medical records of patients aged 18 years or older who underwent surgical treatment for unilateral closed patella fractures at our hospital between August 2016 and August 2020. The incidence of preoperative DVT was detected by Duplex ultrasound (DUS). Partial blood routine and biochemical indexes were collected at admission, and the neutrophil-to-lymphocyte ratio (NLR), monocyte-to-lymphocyte ratio (MLR), and platelet-to-lymphocyte ratio (PLR) of inflammatory indexes were also calculated. ROC was used to analyze the cut-off value NLR, MLR, and PLR for predicting preoperative DVT, and univariate and multivariate analyses of the risk factors for preoperative DVT of patella fractures, and to verify whether other risk factors affecting the relationship between validation indexes and preoperative DVT.

**Results:**

A total of 500 patients were included, of which 39 patients (7.8%) developed preoperative DVT. After univariate and multivariate analysis, preoperative time (in each day delay), male (vs. female), D-dimer > 0.6 mg/L, total cholesterol (TC) > 5.6 mmol/L, and PLR > 189.8 were the risk factors for preoperative DVT in patients with patella fracture. Inflammation index PLR combined with the other four risk factors significantly improved the predictive efficacy of preoperative DVT compared with PLR (*P* = 0.009).

**Conclusion:**

Inflammatory index PLR is a risk factor for preoperative DVT in patients with patella fracture, and the efficacy of PLR in predicting DVT can be significantly improved when other risk factors (male, D-dimer > 0.6 mg/L, TC > 5.6 mmol/L, and PLR > 189.8 of preoperative time in each day delay) are combined. These data are useful for the clinical identification of patients at high risk of preoperative DVT in patella fractures.

## Introduction

DVT is a common complication in patients with lower extremity fractures and carries a risk of fatal pulmonary embolism. DVT has been reported in patients with fractures at the time of admission (1). A patella fracture is a common fracture of the lower extremity with an incidence of 1% to 1.5% (2, 3). Although the incidence is not high, but once the occurrence of activities affecting the knee joint. In addition, the pain after fracture leads to the limitation of lower extremity movement, and the slow venous blood flow caused by immobilization of the affected extremity leads to the risk of DVT (4, 5). Previous studies have shown that the incidence of preoperative DVT in the patella fracture is 4.4% to 5.8% (6, 7).

Understanding the high-risk factors of DVT and early identification of high-risk patients are the key to preventing DVT. However, risk factors are inconsistent in different studies (7–9). Recent studies have found that inflammation is involved in the formation of DVT. Post-traumatic inflammatory response activates inflammatory cells and releases inflammatory factors in the body (10), which leads to systemic reaction and disorder of coagulation and anticoagulation mechanism (11). The neutrophil-to-lymphocyte ratio (NLR), monocyte/lymphocyte (MLR), and platelet to lymphocyte ratio (PLR) are brand new biomarkers of the inflammatory response, which can reflect the overall level of inflammation in the body (12–14). In DVT-related studies, NLR is a high-risk factor for death due to acute DVT in cancer patients (15). Peng et al. (16) found that MLR increased in patients with postoperative venous thromboembolism in patients with hip fractures. Moreover, Kuplay et al. (17) also found that PLR in cardiovascular patients increased significantly in proximal DVT. However, at present, it is not clear whether inflammation indicators NLR, MLR, and PLR are related to the occurrence of preoperative DVT after a patella fracture. And that pushed us to explore.

Our objective was to collect data from patients with surgically treated patella fractures in our hospital and analyze whether inflammatory indicators were risk factors for DVT. In addition, when inflammatory indicators are combined with other risk factors, whether the association between inflammatory indicators and DVT is affected.

## Methods

### Patients

This study retrospectively collected the data of patients with surgically treated patella fracture admitted to our hospital from August 2016 to August 2020. This study was approved by the Ethics Committee of the Hebei Cangzhou Hospital of Integrated Traditional Chinese Medicine and Western Medicine, focusing only on the examination results of patients. All patients enrolled signed informed consent forms. All data are obtained through our electronic medical records system. According to the empirical criteria, the sample size of logistic regression analysis should be 10 to 15 times the number of covariates. There were 36 covariates in this study, and the sample size should not be less than 36 × 10 = 360.

### Inclusion and exclusion

Inclusion criteria were: patients' age to be 18 years old or older, unilateral patella fracture, and surgical treatment. Exclusion criteria: time >2 weeks from fracture, complicated fractures at other sites, open fractures, pathological fractures, severe autoimmune diseases, infectious diseases at the time of fracture, abnormal coagulation function, use of anticoagulants within 3 months of admission, incomplete medical records.

### Data collection

The data collected mainly included the following aspects: demographic data, comorbidities, injuries, blood routine and biochemical test results, and routine DUS test results of DVT.

The demographic data include sex, age, body mass index (BMI), current smoking, alcohol consumption, American Society of Anesthesiologists (ASA). The comorbidities included hypertension, diabetes, chronic heart disease, cerebrovascular disease, and allergies to any medications, all of which were self-reported by patients. Injury-related data included the following: time from injury to hospital admission, mechanism of injury (low or high energy), time from hospital admission to operation, and total length of stay. Low energy injury refers to a fall at a high level while the patient is standing. High energy injury refers to injury caused by traffic accident or position injury higher than their own height.

### Blood collection and test results information

Venous blood was collected from the elbow veins on an empty stomach for the first time after admission.Vein blood samples were collected in vacuum tubes containing sodium citrate anticoagulant. Blood samples were sent to the laboratory of our hospital for blood analysis within 6 h after collection. Laboratory tests included measurements of levels of total protein (TP), albumin (ALB), alkaline phosphatase (ALP), total bilirubin (TBIL), *γ*-glutamyl transferase (GGT), hypersensitive C-reactive protein (HCRP), total bile acid (TBA), creatine kinase (CK), lactic dehydrogenases (LDH), total cholesterol (TC), triglycerides (TG), high-density lipoprotein (HDL-C) level, low-density lipoprotein (LDL-C) level, very low-density lipoprotein (VLDL) level, glucose (GLU), uric acid (UA), white blood cell (WBC) count, neutrophile (NEUT) count, lymphocyte (LYM) count, monocytes (MON) count, red blood cell (RBC) count, hemoglobin (HGB) level, platelet (PLT) count, D-dimer level. Neutrophile-to-lymphocyte rate (NLR), monocytes-to-lymphocyte rate (MLR), platelet-to-lymphocyte rate (PLR) were calculated according to blood routine indexes.

### Diagnosis and prevention of DVT

The patient could not perform off-bed weightbearing exercises because of pain in the injured extremity, but lay in bed until surgery. After admission, all patients were given Low Molecular Weight Heparin Injection (LMWHI) subcutaneous (abdominal subcutaneous) for anticoagulation (4250 I.U., once daily). In addition to drug prophylaxis, the patient underwent physical prophylaxis for DVT with ankle pump exercise in bed (1,000 times, once daily). Drug anticoagulation was discontinued on the day of operation. Patients were screened for DVT by DUS on the first day after admission and every 3 days thereafter, up to the day of operation. If any symptoms of thrombosis (e.g., lower extremity distention, pain, and acute varicose superficial veins) occur in the preoperative time, DUS should be performed immediately to confirm DVT. The occurrence of DVT in patients was detected by the same group of senior ultrasound doctors in our hospital. The “Guidelines for the Diagnosis and Treatment of Deep Vein Thrombosis (2016 3rd Edition)” issued by the Chinese Medical Association was used to diagnose and treat DVTs (18). The veins examined include: femoral common vein, superficial and deep femoral vein, popliteal vein, posterior and anterior tibial vein and peroneal vein. Patients diagnosed with DVT are consulted by vascular surgeons to determine the treatment of thrombosis. The timing of the patient's operation is determined by a senior orthopedic surgeon. The observation period was from the time of fracture to the time of operation, and the time of DVT the first detected by DUS was recorded. Patients were divided into DVT group and non-DVT group according to whether preoperative DVT occurred.

### Statistical analysis

SPSS 25.0 (IBM, Armonk, New York) and MedCalc 20.0 (MedCalc Software, Ostend, Belgium) was used for statistical analysis. For biomarker NLR, PLR, MLR, and the plasma D-dimer level, we constructed receiver operating characteristic (ROC) to determine the optimal cutoff value for each variable. On basis of the cutoff values determined, each variable was divided in to two groups. Categorical variables were analyzed by chi-square or Fisher's exact test. Student T test or Mann-Whitney test was performed for continuous variables. Univariate analysis of differences between DVT group and non-DVT group. Multivariate logistic regression model and stepwise backward elimination method were used to investigate the independent risk factors that predicted the occurrence of DVT. The univariate analysis results of *P* < 0.10 were included in the model, and the correlation strength was expressed by odds ratio (OR) and 95% confidence interval (95% CI). Hosmer–Lemeshow test was employed to evaluate the fitting degree of the final model, and a *P* > 0.05 represented the acceptable result. The ability of inflammatory indicators combined with other independent risk factors to predict preoperative DVT was evaluated by ROC curve again. The combination of indicators and individual inflammatory indicators were compared to predict whether there was statistical difference in preoperative DVT. The significance level was set as *P* < 0.05.

## Results

In the window period, a total of 612 cases of patella fracture patients. According to the exclusion criteria, fracture over 2 weeks; complicated fractures at other sites, open fractures, a total of 54 cases, pathological fracture, severe autoimmune diseases and infectious diseases at the time of fracture, use of anticoagulants within 3 months of admission, a total of 32 cases, 26 cases of incomplete medical records. A total of 500 patients were included.

They were divided into two groups based on the presence or absence of preoperative DVT: 461 patients in non-DVT group and 39 patients in DVT group, respectively. In the DVT group, there were 32 (82.1%) males and 7 (17.9%) females, with an average age of 57.0 ± 15.3 years (range, 28–84 years). In the non-DVT group, there were 273 (59.2%) males and 188 (40.8%) females, with an average age of 52.1 ± 14.9 years (range, 18–86 years). There were 275 (55%) left patella fractures and 225 (45%) right patella fractures. The mean days from fracture to admission was 0.2 ± 0.4 days (range, 0.02 to 5 days), the mean days from fracture to operation was 4.1 ± 2.1 days (range, 0–14 days), and mean total hospital stay was 11.3 ± 3.8 days (rang, 5–26 days).

### DVT patient information

**Figure 1:**
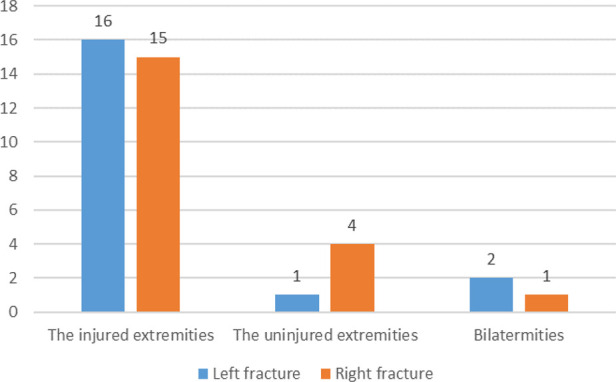
The distribution of DVT in the lower extremity.

**Figure 2:**
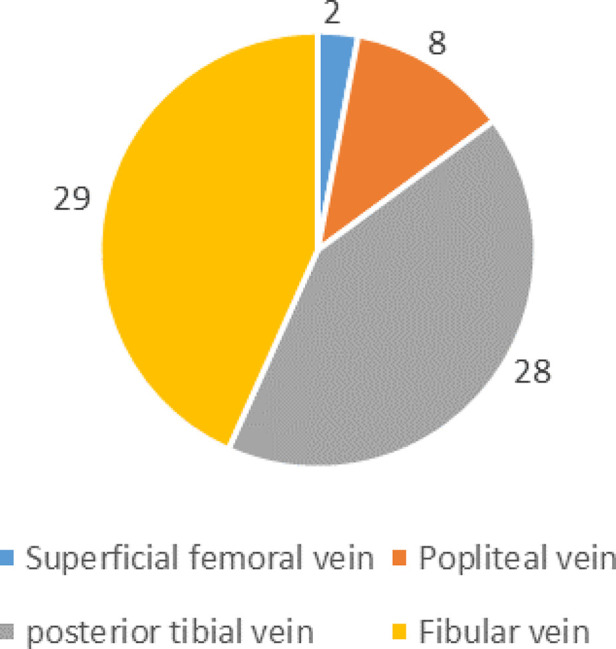
Distribution of DVT in deep veins of the lower extremity.

Preoperative DVT was detected in 39 patients 7.8% (39/500). None of the patients had pulmonary embolism. 79.5% (31/39) occurred in the injured lower extremity and 20.5% (8/39) in the uninjuried lower extremity, as shown in Figure 1. Among all the thrombus, DVT was found in only 4 deep veins, namely superficial femoral vein, popliteal vein, posterior tibial meridians and fibular vein. Their specific distribution is shown in Figure 2. There were 67 clots in 39 patients, with an average of 1.7 clots each patient, and 17 patients had only 1 clots. The mean time from fracture to DVT detection was 3.5 ± 2.2 days (range 0.1–11.1 days).

### ROC analysis of inflammatory indicators for the diagnosis of DVT optimal cutoff value

**Figure 3:**
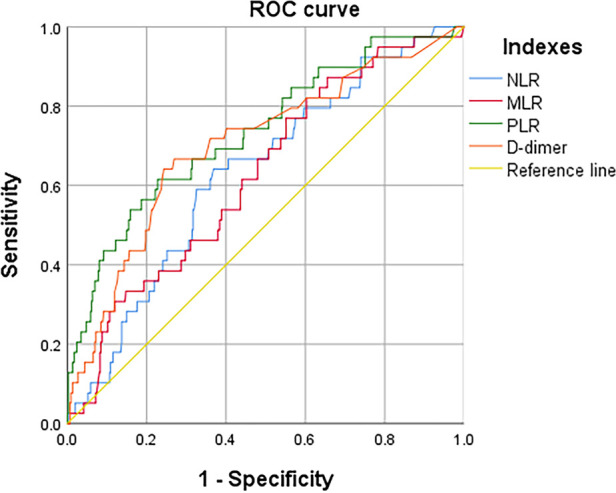
ROC to determine the optimal cutoff value for NLR, LMR, PLR, and D-dimer, when in continuous variable.

According to the ROC curve (Figure 3), the optimal cut off values for predicting DVT of continuous variables NLR, MLR, PLR and D-dimer are selected as follows: NLR, 4.6, AUC, 0.632; MLR, 0.7, AUC, 0.625; PLR, 189.8, AUC, 0.733; D-dimer, 0.6, AUC, 0.701 (as follow Table 1). These inflammatory biomarkers were converted into dichotomous variables based on thresholds.

**Table 1:** The ROC and AUC to determine the optimal cutoff value for each index in continuous variable.

Variable	Optimal cutoff value	AUC	Sensitivity	Specificity	*P* value
NLR	4.6	0.632	0.641	0.631	0.006
MLR	0.7	0.625	0.769	0.449	0.010
PLR	189.8	0.733	0.615	0.772	<0.001
D-dimer	0.6	0.701	0.641	0.757	<0.001

### Univariate analysis of DVT group and non-DVT group

As shown in Table 2, we found statistically that gender, time from fracture to operation, TP < 60 g/L, LDH > 250 U/L, TC > 5.2 mmol/L, PLT > 300*109/L, NLR > 4.6, MLR > 0.7, PLR > 189.8, and D-dimer > 0.6 mg/L was significantly different between DVT group and non-DVT group (*P* < 0.05).

**Table 2:** Univariate analyses of risk factors associated with preoperative DVT after patella fracture.

Variables	Number (%) of DVT (*n* = 39)	Number (%) of non-DVT (*n* = 461)	*P* value
Gender			0.005
Male	32 (82.1)	273 (59.2)	
Female	7 (17.9)	188 (40.8)	
Age (years)	57.0 ± 15.3	52.1 ± 14.9	0.050
BMI (kg/m^2^)	22.9 ± 2.5	24.2 ± 3.2	0.235
Diabetes	4 (10.3)	61 (13.2)	0.596
Hypertension	8 (20.5)	105 (22.8)	0.746
Chronic heart disease	11 (28.2)	114 (24.7)	0.630
Allergy to any medications	3 (7.7)	61 (13.2)	0.320
cerebrovascular disease	4 (10.3)	37 (8.0)	0.626
Mechanism (high-energy)	7 (17.9)	74 (12.1)	0.283
Smoking	8 (20.5)	54 (11.7)	0.109
Alcohol	5 (12.8)	37 (8.0)	0.300
ASA			0.442
I - II	33 (84.6)	409 (88.7)	
III - IV	6 (15.4)	52 (11.3)	
Time from fracture to operation (days)	5.8 ± 1.9	4.1 ± 2.2	<0.001
TP (<60 g/L)	27 (69.2)	239 (51.8)	0.037
ALB (<35 g/L)	19 (48.7)	162 (35.1)	0.090
ALP (>upper limit)	2 (5.1)	34 (7.4)	0.602
TBIL (>upper limit)	2 (5.1)	19 (4.1)	0.763
GGT (>upper limit)	5 (12.8)	39 (8.5)	0.356
HCRP (>8 mg/L)	22 (56.4)	188 (40.8)	0.058
TBA (>10 umol/L)	2 (5.1)	37 (8.0)	0.517
CK (> upper limit)	3 (7.7)	29 (6.3)	0.731
LDH (>250 U/L)	6 (15.4)	30 (6.5)	0.039
HBDH (>182 U/L)	4 (10.3)	30 (6.5)	0.372
TC (>5.2 mmol/L)	13 (33.3)	71 (16.5)	0.004
TG (>1.7 mmol/L)	8 (20.5)	90 (19.5)	0.881
HDL-C (<1.1 mmol/L)	14 (35.9)	132 (28.6)	0.338
LDL-C (>3.37 mmol/L)	8 (20.5)	76 (16.5)	0.518
VLDL (>0.78 mmol/L)	8 (20.5)	88 (19.1)	0.828
GLU (>6.1 mmol/L)	18 (46.2)	155 (33.6)	0.114
UA (> upper limit)	3 (7.7)	51 (11.1)	0.515
WBC (>10*10^9^/L)	13 (33.3)	110 (23.9)	0.187
NEUT (>6.3*10^9^/L)	22 (56.4)	190 (41.2)	0.065
LYM (<1.1*10^9^/L)	11 (28.2)	94 (20.4)	0.250
MON (>0.6*10^9^/L)	24 (61.5)	246 (53.4)	0.325
RBC < lower limit	15 (38.5)	113 (24.5)	0.055
HGB < lower limit	9 (23.1)	87 (18.9)	0.522
PLT (>300*10^9^/L)	17 (43.6)	120 (26.0)	0.018
NLR (>4.6)	24 (61.5)	166 (36.0)	0.002
MLR (>0.7)	13 (33.3)	79 (17.1)	0.012
PLR (>189.8)	23 (59.0)	106 (23.0)	<0.001
D-dimer (>0.6 mg/L)	22 (56.4)	104 (22.6)	<0.001

ALP, alkaline phosphatase, reference range: female, 35–100 U/L; male, 45–125 U/L; TBIL, total bilirubin, reference range: female, 0–21 umol/L; male, 0–26 umol/L; GGT, *γ*-glutamyl transferase, reference range: female, 0–21 umol/L; male, 7–45 U/L, 10–60 U/L; CK, creatine kinase, reference range: female, 40–200 U/L; male, 50–310 U/L; UA, uric acid, reference range: female, 155–357 umol/L; male, 208–428 umol/L; RBC, red blood cell, reference range: Female, 3.5–5.0*1012/L; male, 4.0–5.5*1012/L; HGB, hemoglobin, reference range: Female, 110–150 g/L; male, 120–160 g/L

Multivariate analysis predicted the independent risk factors for preoperative DVT and compared the differences after combination.

**Figure 4:**
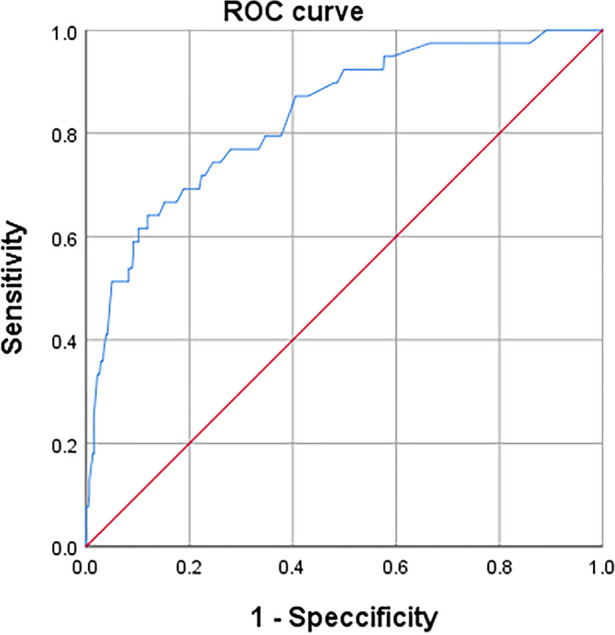
The ROC curve of the combined diagnostic index A for the diagnosis of preoperative DVT.

Multivariate logistic regression model analysis found five independent risk factors for preoperative DVT, which were male(vs. female) (*P* = 0.008), time from fracture to operation (in each day delay) (*P* < 0.001), D-dimer > 0.6 mg/L (*P* < 0.001), TC > 5.2 mmol/L (*P* = 0.005), and PLR > 189.8 (*P* < 0.001), as shown in Table 3. The H-L test of demonstrated The good fitness of The final model (X2 = 5.680, *P* = 0.683; Nagelkerke R2 = 0.289). After PLR was combined with other 4 factors, it was found that AUC, sensitivity and specificity for predicting preoperative DVT were 0.833, 0.641 and 0.883 (Figure 4). Better than PLR alone in predicting DVT efficacy (AUC, 0.833 vs. 0.733, *P* = 0.009).

**Table 3:** Multivariate analysis of factors associated with preoperative DVT of patella fractures.

Variables	OR and 95%CI	*P* value
Gender (male vs. female)	3.53 (1.39–8.92)	0.008
Time from fracture to operation (in each day delay)	1.31 (1.12–1.52)	<0.001
D-dimer (>0.6 mg/L)	3.78 (1.81–7.89)	<0.001
TC (>5.2 mmol/L)	1.80 (1.20–2.70)	0.005
PLR (>189.8)	3.73 (1.80–7.77)	<0.001

## Discussion

Patients with patella fracture were selected for a retrospective analysis of the occurrence of preoperative DVT and related risk factors, and the risk factors were linearized to predict the occurrence of DVT again. In this study, we found that the incidence of preoperative DVT was 7.8%, and identified male, time from fracture to operation (in each day delay), D-dimer > 0.6 mg/L (*P* < 0.001), TC > 5.2 mmol/L (*P* = 0.005), and PLR > 189.8 were risks factor for preoperative DVT in patients with patella fracture. The AUC of preoperative DVT after patella fracture predicted by ROC analysis after PLR combined with other 4 risk factors was 0.833, the sensitivity was 0.883, and the specificity was 0.641.

Data from this study showed that the incidence of preoperative DVT was 7.8% in patients with patella fractures. Previous studies have shown that the incidence of preoperative DVT is between 4.1% to 52.5% in patients with perioperative knee fracture, including fractures of the distal femur, patella, and proximal tibia (6, 7, 19–21). The incidence of DVT in the perioperative period of preoperative patella fracture was 4.1%–5.8% (6, 7), and the results of this study were basically consistent with them. In terms of location, 79.5% of DVT occurred in the affected lower extremities, and 85% of DVT occurred in the posterior tibial vein and fibular vein, which was consistent with Yang et al. (6) and Luo et al. (22).

In this study, male was a risk factor for the occurrence of preoperative DVT in patients, and the risk of male was 2.53 times higher than that of female. In other studies, the risk of DVT in males is 1.29–2.35 times higher than that in females (6, 18, 23). However, females in childbearing age are higher than males, while males are higher than females in non-childbearing age (24, 25). In our sample, 66.7% (26/39) of the DVT group were over 50 years old, which was consistent with the epidemiological statistical results. Some reports indicate that the difference may be related to hormone levels and genetics in women (24, 26, 27). The results of this study also showed that for each additional day before surgery, the risk of developing preoperative DVT increased by 31%. It's also an easy ending to understand. The patient's bed rest after fracture slows down the blood velocity of the lower extremities. On the other hand, the hypercoagulability of blood after trauma contributes to the occurrence of DVT, which also conforms to Virchow's Law (28). Similar results have been confirmed in spinal fracture (29), hip fracture (30), patella fracture (6) and foot fracture (8). With the increase of preoperative time, the risk of DVT in patients is 5% to 29%.

D-dimer is a recognized diagnostic index for the occurrence of DVT, which reflects the coagulation and fibrinolysis state of the body (31–33). Our results also show this characteristic of D-dimer. There is evidence that the D-dimer threshold for DVT diagnosis in patients with spinal and tibial fractures is 1.08 mg/L and 1.76 mg/L, respectively (21, 34). In this study, the threshold was 0.6 mg/L by ROC analysis. In terms of risk, we found that patients with D-dimer > 0.6 mg/L were 3.78 times more likely to develop DVT than patients with D-dimer ≤ 0.6 mg/L. It is well known that high plasma TC levels increase the incidence of cardiovascular events and death (35). We found that TC > 5.2 mmol/L increased the incidence of preoperative DVT, and multivariate analysis showed that the risk of DVT increased by 0.8 times. Leiba et al. (36) found that patients with high cholesterol significantly increased in patients with DVT, which was consistent with our research results.

Although NLR and MLR univariate analyses showed differences between the DVT and non-DVT groups. However, multivariate analysis did not identify an independent risk factor for preoperative DVT. We found that the AUC of PLR predicting DVT at admission was 0.733, and the sensitivity and specificity were 0.615 and 0.772, respectively. Patients with PLR > 189.8 had a 2.73-fold increased risk of preoperative DVT. Systemic inflammatory response and immunosuppression can be triggered immediately after trauma (37). Platelets, as cellular effector factors of inflammation and thrombosis, can also be activated after trauma (38). It may stimulate this inflammatory response *in vivo* and cause the increase of platelets (39). There is evidence that platelets play an important role in DVT (40). Multiple studies have shown that high PLR is associated with DVT (41–43), and there is also evidence that high PLR is associated with acute DVT (15). Other studies have shown that high PLR increases the risk of venous thrombosis by nearly 3 times (43). The results of this study are consistent with those reported.

Yao et al. (42) found that patients with postoperative DVT had higher preoperative PLR, but lower sensitivity and specificity in predicting DVT. We found similar results. Independent PLR showed low efficacy and low sensitivity and specificity in predicting preoperative DVT (AUC, 0.733, sensitivity, 0.615, specificity, 0.772). We linearized the combination of inflammatory indicator PLR with other 4 independent risk factors at admission, and found that the AUC for predicting DVT was 0.834, and the sensitivity and specificity were 0.641 and 0.839, respectively, which further proved that PLR after combination was highly correlated with the occurrence of preoperative DVT. The predictive efficacy and specificity of DVT were improved (*P* = 0.009). However, the sensitivity is not high, only 0.641. We analyzed that PLR may be affected by other factors, but this needs further confirmation.

There are some limitations of this study: First, we did not collect anticoagulant and blood drawing time after admission and before fasting venous blood drawing. Second, the amount of intravenous fluids in different patients before surgery may not be completely consistent. Third, none of the patients moved out of bed before surgery. These causes may influence independent factor results or the incidence of DVT.

## Conclusions

By collecting data from patients with patella fractures, it was found that the incidence of preoperative DVT for patella fractures was 7.8%. Multivariate analysis showed that male, preoperative time (in each day delay), TC > 5.2 mmol/L, D-dimer > 0.6 mg/L, and PLR > 189.8 were independent risk factors for preoperative DVT of patella fracture. PLR combined with other risk factors improved the ability to predict preoperative DVT. Better than PLR alone (*P* = 0.009). PLR is expected to be a predictor of DVT, especially when other risk factors are combined.

## Data Availability

The original contributions presented in the study are included in the article/Supplementary Material, further inquiries can be directed to the corresponding author/s.
